# Enhancing patient care: pharmacotherapeutic monitoring in community pharmacies – a literature review

**DOI:** 10.1097/MS9.0000000000003039

**Published:** 2025-02-06

**Authors:** Samuel Inshutiyimana, Laura Ghanem, Nagham Ramadan, Doha Hammoud, Magda Wojtara, Sarah Mshaymesh, Olivier Uwishema

**Affiliations:** a Department of Research and Education, Oli Health Magazine Organization, Kigali, Rwanda; b Department of Pharmaceutics and Pharmacy Practice, School of Pharmacy and Health Sciences, United States International University-Africa, Nairobi, Kenya; c Faculty of Medical Sciences, Lebanese University, Beirut, Lebanon; d Department of Medicine, Faculty of Medicine, Beirut Arab University, Beirut, Lebanon; e Department of Clinical Pharmacy, National University of Pharmacy, Kharkiv, Ukraine; f Department of Human Genetics, University of Michigan Medical School, Ann Arbor, Michigan; g Division of Natural Sciences, Faculty of Sciences, Haigazian University, Beirut, Lebanon

**Keywords:** adverse drug effects, adverse events, community pharmacy, patient care, pharmacotherapeutic monitoring, therapeutic drug monitoring

## Abstract

**Background:**

The role of community pharmacies has been transitioning from traditional drug dispensing methods to include more patient-centered services such as pharmacotherapeutic monitoring. With the increasing complexity of medication regimens and the rising prevalence of chronic diseases, pharmacotherapeutic monitoring plays a crucial role in preventing medication-related problems. This study aims to review patient outcomes of community pharmacies’ involvement in pharmacotherapeutic monitoring services. It also highlights advances in the monitoring process, the challenges involved, and relevant solutions.

**Methods:**

A narrative review was conducted from 8 April 2024 to 9 May 2024, where data were extracted from multiple research studies indexed in PubMed, EBSCOHost, and Google Scholar databases. This review included only previous articles written in English and had information relevant to community pharmacy, patient care, innovations in pharmacotherapeutic monitoring, and challenges associated with the monitoring process in community pharmacies.

**Results:**

Implementation of pharmacotherapeutic monitoring in community pharmacies improves patient outcomes and satisfaction. It minimizes adverse events by ensuring optimal dosing and patient adherence, which in turn reduces medical expenses and duration of hospitalization. However, factors such as inefficient analytical resources, lack of skilled personnel, and huge financial costs complicate implementation of pharmacotherapeutic monitoring in community pharmacies.

**Conclusion:**

Community pharmacies’ participation in therapeutic drug monitoring is a promising development that prioritizes tailored patient care, eliminating the need for hospital visits. We recommend a multidisciplinary collaboration, robust electronic medical record systems, and large-scale research studies to provide sufficient evidence on the application of therapeutic drug monitoring in community pharmacies, to leverage this service. Also, policy and decision-makers should facilitate this practice by investing more in pharmacotherapeutic monitoring tools and training for community pharmacists.

## Introduction

Therapeutic drug monitoring (TDM), also known as pharmacotherapeutic monitoring is defined as a healthcare operation that involves testing the dosage of medications, aiming to adjust and optimize their concentration and bioavailability in a patient’s blood circulation^[1]^. However, this strategy is not necessarily considered for all medications. It is only applied when a drug has a narrow therapeutic window, a complicated pharmacokinetic metabolism, and a wide inconsistency in its bioavailability. Moreover, TDM is applied when it is difficult to monitor the targeted dosage or the drug is associated with a risk of toxicity to the tissues of the body^[1]^. Many factors such as age, race, severity of illness, and drug–drug reactions, among others, can influence plasma concentration of a certain prescribed drug^[1,2]^. It is worth noting that the pharmacological field has been widely developed over the past few decades, and is now considered as an essential sector in the healthcare system^[3,4]^. Pharmacists can now assist in various manufacturing processes, including drug formulation, quality analysis, mixing the different pharmaceutical compounds, and the release of the medications to the market^[3]^. It has been shown that patients who take physician-prescribed mediations are at high risk of adverse effects at any time, due to various factors^[4,5]^. Therefore, it is essential to adopt an enhanced interprofessional collaboration among physicians and pharmacists in order to minimize drug toxicity, especially in the case of chronic diseases^[4,5]^. This review elaborates on the patient outcomes of pharmacotherapeutic monitoring in community pharmacies. Furthermore, it highlights advances in the monitoring process, challenges present, and suitable solutions.

**Highlights**

- Pharmacotherapeutic monitoring is crucial for preventing medication-related problems, given complex medication regimens and rising chronic diseases.
- Challenges include inefficient analytical resources, lack of skilled personnel, and high financial costs.
- Governments and healthcare organizations should prioritize and support the development and incorporation of therapeutic monitoring services in community pharmacies to enhance patient care.
- Recommendations for improvement include multidisciplinary collaboration, robust electronic medical record systems, and large-scale research studies.

### Role of community pharmacies in patient care

**Figure 1.**
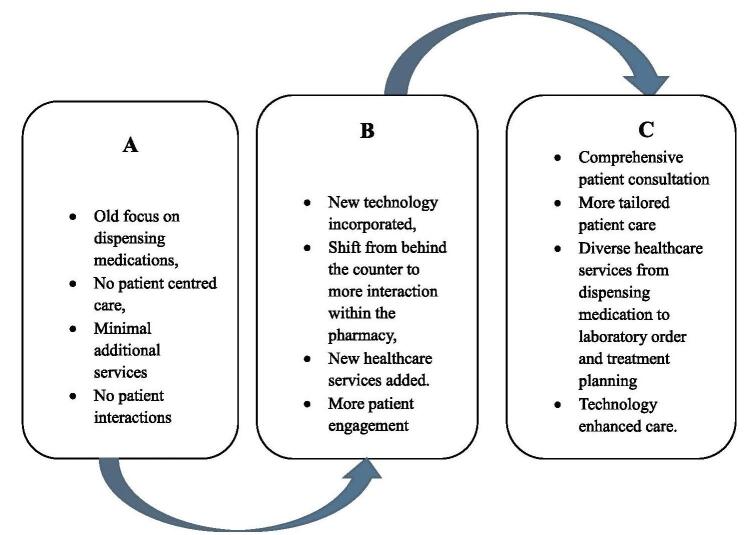
Evolution of pharmacy practice: from medication dispensing to patient centered approach. A: Traditional pharmacy or product centered, B: Evolution, C: Developed pharmacy or patient-centered.

Community pharmacies are underutilized resources which facilitate access to patient care whenever and wherever patients need it. There is narrative that community pharmacies primarily focus on medication dispensing and selling. However, various types of services are offered by community pharmacies depending on the country or state of jurisdiction. Community pharmacies are transforming healthcare by providing emergency medication refills, initiating new prescription drugs, ordering, and interpreting laboratory results^[6]^. Countries such as the United States of America (USA) and Switzerland have installed several policies to expand the uses and roles of community pharmacies to facilitate patient coordinated care delivery. Despite all the restraints on the sector, community pharmacies in the mentioned countries have evolved beyond medication prescriptions. Services have now been divided to involve multiple areas such as medication optimization, wellness and prevention, chronic and acute care management, and patient education, among others^[7]^. Therefore, it is important to mention the need of including patient-centered care in community pharmacy in multiple communities. This care requires shared decision-making and satisfaction, prioritizing the patient’**s** values, preferences, and needs over those of the provider or organization^[8]^. Patient-centered care is important as a way to enhance patient satisfaction by meeting patient expectations and more^[8]^. Patient satisfaction is influenced by personal expectations rather than healthcare quality and can be affected by various factors, such as inpatient services, prescriptions, healthcare expenditure, and mortality risk^[9]^. Incorporating patient-centered care in community pharmacies will allow for more tailored care to each individual patient’s specific needs, thus improving healthcare outcomes and increasing medication adherence^[10]^. Overall, a patient-centered approach has promoted patient well-being, preventive care, and reduced potential healthcare costs. Consequently, this has the potential to decrease patient burden and encourage patients to seek medical help more frequently^[11]^ (see Fig. 1).

## Methodology

A narrative review was thoroughly conducted from 8 April 2024 to 9 May 2024. PubMed, Google Scholar, EBSCOHost searches were employed to retrieve peer-reviewed articles. The search strategy included the use key terms: “pharmacotherapeutic monitoring,” “community pharmacy,” “patient care,” “medication therapy management,” “pharmacist interventions,” and “medication adherence.” Such terms were combined using Boolean operators (AND, OR) to maximize the breadth of relevant studies. The articles included were those written in English, discussing community pharmacies with access to pharmacotherapeutic monitoring services, and examining the impact of monitoring on patient outcomes, as well as the advances and challenges associated with the monitoring process. The search excluded studies done in hospital or other healthcare settings that do not involve community pharmacies. The selection process was conducted in two stages. The first step involved the identification of titles and abstracts that were screened for relevance by different reviewers. Any uncertainty about an article’s eligibility was solved through the revision of the full text. The second step involved the assessment of full-text articles following the inclusion-based criteria. In the preparation of the final manuscript, all collated articles were further cross-checked to ensure their relevance to the aim of this review.

## Discussion

### Overview of pharmacotherapeutic monitoring process

Pharmacotherapeutic monitoring plays a critical role in enhancing the patient’s quality of life by ensuring the most effective treatment with minimal side effects^[1,2]^. However, a fundamental step before starting the monitoring process is to check whether a medication is suitable for examination and inspection^[12]^. To ensure a successful therapeutic effect, it is essential to check the normal concentration, the required concentration, the approach to adjusting the specific agents in the medication, and whether the evidence supports applying TDM to the specific medication^[12]^. Once these concerns are addressed, the TDM can be applied efficiently to prevent underdosing, which can compromise the therapeutic effect of the drug, and overdosing, which can put the patient at risk of toxicity and morbidity^[12]^. The determination of this depends on many factors, including the patient’s age, ethnicity, disease stage and severity, drug interactions, and previous antimicrobial resistance, among others, with the goal of achieving successful illness management^[1,2]^. It is important to note that the TDM approach consists of many principles such as efficacy, adherence, drug interactions, adverse toxic effects, and monitoring during treatment and after termination of medication regimen^[1]^.

**Figure 2.**
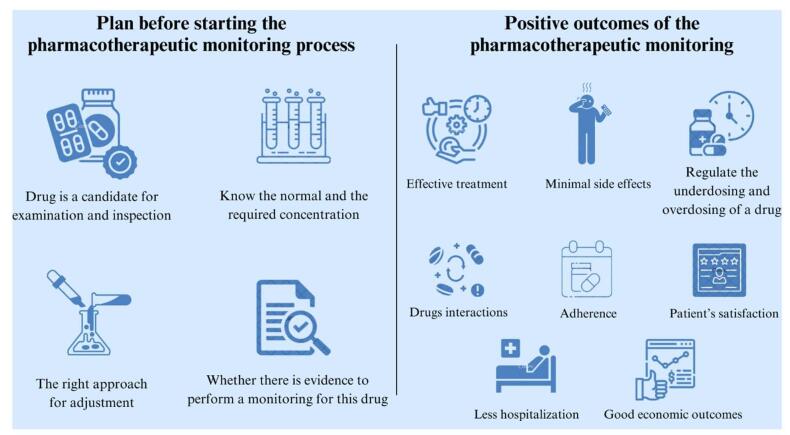
Elements of a pharmacotherapeutic monitoring plan and the positive outcomes of the monitoring process.

A study conducted to highlight a follow-up of pediatric cancer medication revealed the need of specific intrusions in 25% of the patients^[13]^. Moreover, 32 drug-related problems (DRPs) were noted, recommending the demand of “necessity,” “safety,” and “effectiveness”^[13]^. This highlights the importance of drug monitoring in children during the cancer treatment^[13]^. Another study shed light on the patient’s satisfaction from the services such as drug monitoring, provided by pharmacists^[14]^. Patients are satisfied when the pharmacists provide more knowledge about drugs and compliance. Additionally, effective communication between patients and prescribers was highlighted as an essential factor in patient’s gratification^[14]^. Moreover, TDM and other pharmaceutical services played a crucial role in improving both clinical and economic outcomes by reducing medication errors and hospitalizations, leading to positive economic outcomes^[1,11]^. (See Fig. 2)

The main obstacle to implementing an effective monitoring process is the inefficiency of analytical methods^[12]^. Modern methods like liquid chromatography coupled with mass spectrometry have significantly enhanced the accuracy and sensitivity of TDM, but these powerful technologies often require large, specialized laboratories and rely on central remote facilities for analysis, leading to significant delays due to the need for sample postage^[12]^. This delay in processing the samples can be detrimental to the patient care, particularly when timely adjustments to drug dosages are necessary^[12]^. Moreover, inadequate resources in community pharmacies, essentially computers convenient for all the practitioners to discuss emerging pharmacotherapeutic advice continue to challenge the process^[12]^. Gathering all the necessary information from patients’ blood samples for adequate analysis has become more difficult for scientists^[12]^. Another barrier is the financial costs that patients might have to bear^[12,15]^.

### Advances in pharmacotherapeutic monitoring

The development of innovative methods and technology has driven advances in pharmacotherapeutic monitoring. These cover a wide range of options, from computerized medication delivery systems to wearable sensors and mobile applications. Electronic medicine distribution systems facilitate the dispensing process by incorporating features like dosage tracking and pharmaceutical inventory management. Wearable sensors enable constant tracking of physiological indicators, delivering real-time information about a patient’s health status. Medication reminders with features such as dose scheduling, refill reminders, and drug interaction alerts improve medication adherence by 15–25% through personalized reminders. Moreover, they improve medication safety by decreasing adverse drug reactions by 25%^[16,17]^. These tools give healthcare clinicians with actionable information into their patients’ medication use patterns, allowing for more personalized interventions and better therapeutic outcomes^[18,19]^. The use of precision medicine approaches, such as pharmacogenomics, has also shown promise in optimizing drug therapy. Tailored interventions based on individual genetic profiles have been linked to a 40% increase in therapeutic efficacy and a significant reduction in adverse reactions, particularly in patients receiving treatment for cardiovascular diseases and cancer^[20]^.

Furthermore, innovative software programs play a critical role in improving drug management procedures. Prescription reminder applications, such as Medisafe and Mango Health, use simple interfaces and customizable features to assist patients stick to their prescription schedules. These apps give dose reminders, refill notifications, and educational information on drug use. Reports indicate that these applications have achieved adherence improvements of 20–35% in patients with chronic conditions^[16,17]^. Additionally, medication reconciliation software, such as Surescripts and DrFirst, simplifies the medication reconciliation procedure across healthcare settings, lowering medication inconsistencies and adverse drug reactions. Electronic health record (EHR) systems with integrated medication monitoring features, such as Epic and Cerner, allow for seamless documentation and transmission of drug-related information across healthcare practitioners, improving care coordination and patient safety^[21]^. These systems have been associated with a 25% reduction in adverse drug events and a 30% improvement in medication adherence due to their ability to track patient medication histories and identify potential drug interactions^[8,22]^.

The incorporation of technology into community pharmacy processes has transformed traditional methods while improving patient care delivery^[22]^. Automated dispensing systems, including ScriptPro and Omnicell, improve prescription fulfillment operations by lowering wait times and pharmaceutical errors. Medication synchronization programs, such as Simplify My Meds and PrescribeWellness, encourage medication adherence by coordinating refill schedules and lowering barriers to medicine access. Telepharmacy programs, such as PipelineRx and TelePharm, bring pharmacy services to impoverished communities, improving prescription access and patient outcomes. Remote medication monitoring technologies, such as AdhereTech and AiCure, allow chemists to remotely monitor patients’ medication adherence and engage proactively to resolve adherence hurdles, resulting in improved medication adherence and clinical outcomes^[23-25]^. For instance, telepharmacy programs have reduced medication errors by up to 35% and improved adherence rates by 15–20% through virtual consultations and remote monitoring^[5,7]^ (see Table 1).

**Table 1:** Different tools used in pharmacotherapeutic monitoring

Advanced monitoring tools	Features	Usability	Effectiveness	Type of study	Number of participants	References
Electronic Medication	Automated dispensing, dosage tracking	User-friendly	Improve medication adherence up to 30%	Observational	Not provided	^[26,27]^
Dispensing Systems	Medication inventory management	With Pharmacy workflows	Prevent medication errors	Observational	Not provided	^[28]^
Smart Pill Bottle	Remotely monitor medication adherence, provide dosage reminders	Intuitive design, connectivity with mobile devices	Improved adherence by 25%–35%, Reduced hospitalizations due to non-adherence by 10–15% in chronic patients	Observational	200	^[16,29]^
Wearable Sensors	Continuous monitoring of physiological parameters	Comfortable to wear, data accuracy	Reduced hospital readmissions by 15%, increased patient satisfaction by 20% due to improved chronic disease management	Mixed Methods	300	^[30]^
Medication Reminder Apps	Dose scheduling, refill reminders, drug interaction alerts	User-friendly interface, customization options	Improve medication adherence by 15–20% through personalized timely reminders and education.	Randomized Clinical Trial and Cohort	400	^[16,17]^
			Decreased adverse drug reactions by 25%			

### Case studies

If an underlying condition, such as kidney failure, is noticed at any point, another measure of the plasma drug concentration can be applied to adjust the dosage accordingly^[1]^. Another alarming factor that can prompt a physician to perform concentration tests is drug toxicity, which can be manifest through various symptoms^[1,17]^. When the patient is taking aminoglycoside antimicrobial drugs, it would be efficient to measure the plasma drug concentration to distinguish between nephrotoxicity caused by the infection or the medication itself^[1,31]^. For example, the toxicity of digoxin, a medication prescribed to manage heart failure, can be identified through signs related to heart disease^[1,32]^. Regarding prophylaxis drugs, the targeted concentration cannot be predicted for prevention, so it is inefficient to take some measurements beforehand^[1]^. However, for drugs with a narrow therapeutic window, such as cyclosporine, lithium, and aminoglycosides antibiotics, other concerns should be considered, and the dosage should be reliable^[1]^.

The implementation of pharmacotherapeutic monitoring strategies has shown several benefits for the patient. One of the main components of this approach is a multidisciplinary collaboration among the healthcare providers, including biologists, physicians, nurses, and pharmacists, aimed at achieving the most efficient practice in this sector^[1,13]^. A robust system for electronic medical records is expected to be essential in optimizing pharmacotherapeutic monitoring^[12]^. It is highly recommended that highly skilled scientists be employed to take responsibility for the monitoring process in laboratories, ensuring high precision for better accuracy and interpretation of the available useful data^[1]^. The results of the assays performed to detect drug toxicity should be out quickly, preferably within 24 hours. Therefore, it is suggested that the implementation of an external quality assurance program can be beneficial^[1]^. As mentioned earlier, since a patient’s characteristics can influence the decision on a convenient dosage, it is crucial to emphasize a thorough interpretation of the plasma drug concentration, taking all these factors into account^[1]^. To develop efficient strategies, the USA introduced precision medicine, an initiative aimed at collecting data on the variability of molecular and biochemical factors to create the most effective preventative and therapeutic medications^[12]^ (see Table 2).

**Table 2:** The presentation of some cases where plasma drug concentrations should be tested during a follow-up, as well as some strategies that were introduced to optimize the TDM

When to measure plasma drug concentration	Implementations to optimize the pharmacotherapeutic monitoring	References
If an underlying cause was noticed	Multidisciplinary approach	^[1]^
In case of drug toxicity	A robust system for electronic medical records	^[1,12]^
To know the etiology of the symptoms that the patient had manifested	The employment of skilled scientists	^[1]^
If the drug has a narrow therapeutic window	The emergence of fast laboratory test results, with a good interpretation	^[1]^
	The implementation of the “precision medicine” program to collect more data	^[12]^

### Regulatory and ethical considerations

A variety of rules govern pharmacotherapeutic monitoring techniques, all of which are intended to ensure patient safety, integrity of information, and legal compliance. The Food and Drug Administration in the United States and the European Medicines Agency in Europe set standards for the progress, marketing, and use of pharmacotherapeutic monitoring systems. These rules include criteria for product quality, clinical validation, and post-market surveillance to reduce the risks of drug errors, adverse events, and data breaches. Furthermore, legislation in the United States, such as the Health Insurance Portability and Accountability Act, oversees the privacy and security of patient health information, imposing stringent data protection and confidentiality requirements^[33]^.

Moreover, ethical considerations are critical in pharmacotherapeutic monitoring, particularly with patient privacy and data management. When collecting, keeping, and processing patient data, healthcare providers and technology developers must adhere to the principles of autonomy, beneficence, and nonmaleficence. Patients have the right to informed consent and oversight over the use of their health information, which necessitates clear communication and strong consent mechanisms. Furthermore, sharing sensitive medical data between healthcare practitioners and third-party businesses may raise ethical concerns, including issues of data security, confidentiality, and potential breaches of trust^[34,35]^.

Regulatory compliance also has a substantial impact on patient care, influencing the adoption, implementation, and effectiveness of pharmacotherapeutic monitoring procedures. Adherence to regulatory requirements assures the safety, efficacy, and quality of monitoring technology, creating trust in healthcare practitioners, patients, and regulators. However, regulatory requirements might pose difficulties in terms of resource allocation, time limits, and administrative costs, potentially affecting the accessibility and affordability of monitoring services. Compliance with laws fosters a culture of accountability, continuous improvement, and patient-centered care, driving innovation and advancing the standards of pharmacotherapeutic monitoring to improve patient outcomes and care delivery ^[36]^.

### Future directions and innovations

Emerging healthcare and technology trends are expected to create significant advancements in pharmacotherapeutic monitoring. One notable trend is the incorporation of artificial intelligence (AI) tools such as machine learning algorithms into monitoring systems, allowing for predictive analytics and personalized interventions. AI-powered solutions can analyze large datasets to find patterns, anticipate pharmaceutical responses, and optimize therapy regimens based on patient characteristics^[20]^. Another trend is the rise of wearable biosensors and mobile health applications, which enable real-time monitoring of physiological indicators and medication adherence outside of clinical settings. Furthermore, the use of remote patient monitoring technology and telehealth services is likely to grow, thereby improving access to care and enabling for proactive management of chronic illnesses through continuous monitoring and virtual consultation^[37-39]^.

Additionally, advances in technology and data analytics have the potential to transform pharmacotherapeutic monitoring methods. Novel sensor technologies, such as implantable microchips and ingestible sensors, provide unprecedented real-time monitoring of compliance with medications and physiological data in the body. These technologies allow for exact dosage modifications and the early detection of adverse drug reactions, opening the door for personalized medicine and precision treatments. Furthermore, innovations in data analytics, such as big data analytics and blockchain technology, provide healthcare stakeholders with safe, interoperable, and actionable insights drawn from a wide range of healthcare data ^[40]^.

**Figure 3.**
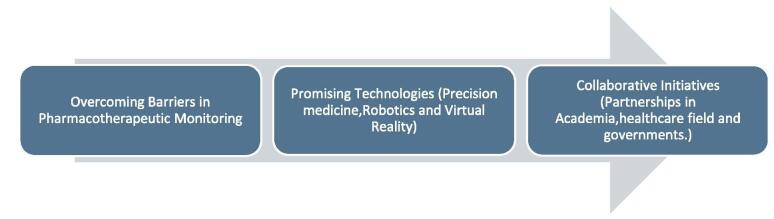
Future directions and innovations in pharmacotherapeutic monitoring.

To realize the full potential of pharmacotherapeutic monitoring technologies, collaboration and interdisciplinary methods are critical. Partnerships between healthcare providers, researchers, technology developers, and regulatory bodies can stimulate innovation, speed technology adoption, and address difficult healthcare concerns. Interdisciplinary research endeavors in pharmacology, engineering, data science, and behavioral science can result in ground-breaking discoveries and creative solutions to improve medication administration and patient care. Furthermore, collaborative networks and consortia promote knowledge sharing, resource pooling, and collaborative problem-solving, allowing stakeholders to harness collective expertise and resources to advance pharmacotherapeutic monitoring methods and achieve meaningful outcomes^[41]^ (see Fig. 3).

## Limitations

Several limitations were identified while developing this review. All studies published in languages other than English were excluded, which presents potential language bias. Moreover, the varied study designs and outcome measures used in the included studies made it challenging to draw definitive conclusions about the effects of pharmacotherapeutic monitoring, such as its impact on minimizing adverse events, improving patient adherence, and reducing medical expenses. Furthermore, the data presented herein cannot be applied to a specific population or country due to the inadequacy of existing literature. Lastly, the pharmacy practice evolution may be a limiting factor to the applicability of older studies to the current practices in community pharmacies.

## Conclusion

Incorporating pharmacotherapeutic monitoring in community pharmacies has been shown to significantly shift pharmacy services towards more patient-centered care. Findings from this review confirm the effectiveness of pharmacotherapeutic monitoring which not only improves patient outcomes but also plays a role in healthcare gratification. It improves medication safety and decreases costs by enhancing the efficacy of drugs and minimizing drug associated complications. Moreover, it enhances patient satisfaction and engagement by guaranteeing tailored care to individuals’ needs. Nevertheless, incorporation of such programs is associated with challenges such as inadequately trained pharmacists, use of emerging technology systems, and overcoming regulatory and financial reimbursement. Therefore, we urge policy and decision makers to prioritize and facilitate the ongoing development and incorporation of pharmacotherapeutic monitoring services in community pharmacies.

Expanding community pharmacy services may be achieved through the establishment of new funding models and improving qualifications of pharmacists. This includes expanding the role of community pharmacies to include health assessment, health promotion, and medication use reviews. Additionally, community pharmacies can benefit by integrating digital health solutions into their practice, collaborating with primary care teams, identifying public health threats through research initiatives, and providing effective and innovative services to reduce the burden on other sectors and services.These actions and innovations will thus allow community pharmacies to flourish and become more oriented towards providing tailored patient care. The adoption of pharmacotherapeutic monitoring in the setting of community pharmacies is a feasible and beneficial investment in the healthcare system. Acting towards the facilitation of these changes is urged to enable pharmacies to meet the diverse needs of patients and significantly improve the overall healthcare system. Collectively, partnerships between healthcare providers, researchers, technology developers, and regulatory bodies can stimulate innovation, speed technology adoption, and address difficult healthcare concerns in community pharmacies.

## Data Availability

Data availability is not applicable to this article as no new data were created or analyzed in this study.
